# Exploring Phylogenetic Signal in Multivariate Phenotypes by Maximizing Blomberg’s *K*

**DOI:** 10.1093/sysbio/syae035

**Published:** 2024-07-06

**Authors:** Philipp Mitteroecker, Michael L Collyer, Dean C Adams

**Affiliations:** Department of Evolutionary Biology, University of Vienna, Djerassiplatz 1, 1030 Vienna, Austria; Konrad Lorenz Institute for Evolution and Cognition Research, Martinstraße 12, 3400, Klosterneuburg, Austria; Department of Science, Chatham University, 107 Woodland Road, Pittsburgh, PA 15232, USA; Department of Ecology, Evolution, and Organismal Biology, Iowa State University, 2200 Osborne Dr, Ames, IA 50011, USA

**Keywords:** Cranial shape, multivariate evolution, phylogenetic generalized least squares, phylogeny, relative eigenanalysis

## Abstract

Due to the hierarchical structure of the tree of life, closely related species often resemble each other more than distantly related species; a pattern termed phylogenetic signal. Numerous univariate statistics have been proposed as measures of phylogenetic signal for single phenotypic traits, but the study of phylogenetic signal for multivariate data, as is common in modern biology, remains challenging. Here, we introduce a new method to explore phylogenetic signal in multivariate phenotypes. Our approach decomposes the data into linear combinations with maximal (or minimal) phylogenetic signal, as measured by Blomberg’s K. The loading vectors of these phylogenetic components or K-components can be biologically interpreted, and scatterplots of the scores can be used as a low-dimensional ordination of the data that maximally (or minimally) preserves phylogenetic signal. We present algebraic and statistical properties, along with 2 new summary statistics, $K_{A}$ and $K_{G}$, of phylogenetic signal in multivariate data. Simulation studies showed that $K_{A}$ and $K_{G}$ have higher statistical power than the previously suggested statistic $K_{m\,u\,l\,t}$, especially if phylogenetic signal is low or concentrated in a few trait dimensions. In 2 empirical applications to vertebrate cranial shape (crocodyliforms and papionins), we found statistically significant phylogenetic signal concentrated in a few trait dimensions. The finding that phylogenetic signal can be highly variable across the dimensions of multivariate phenotypes has important implications for current maximum likelihood approaches to phylogenetic signal in multivariate data.

Phylogenetic signal describes the tendency of closely related species to resemble each other more so than distantly related species. Numerous statistical measures have been proposed as summaries of phylogenetic signal (e.g., Gittleman and Kot, 1990; Pagel, 1999; Freckleton et al., 2002; Blomberg et al., 2003; Felsenstein, 2004; Revell et al., 2008; Collyer et al., 2022), with most being developed for single (univariate) traits. However, biological phenotypes are highly multivariate, and evolutionary biologists are increasingly turning their gaze towards understanding the evolution of these multivariate traits (summarized in: Adams and Collyer, 2018a, 2019; Collyer and Adams, 2021). Unfortunately, for many multivariate datasets, such as those found in modern morphometrics, univariate measures of phylogenetic signal applied to each measured trait separately are inappropriate because biological interpretations are typically based on linear combinations of a large number of variables. Single variables often are not meaningful *per se*. Similarly, the application of univariate measures of phylogenetic signal to the ordinary principal components (PCs) of the variables is ineffective as the PCs just maximize phenotypic variation, not necessarily phylogenetic signal.

Because of this disconnect, a generalization of one measure was proposed (termed $K_{\mathrm{mult}}$) that provides an overall estimate of the degree of phylogenetic signal observed in multivariate datasets (Adams, 2014). A survey of the empirical literature (Adams and Collyer, 2019) revealed that a large majority of multivariate datasets scrutinized with $K_{\mathrm{mult}}$ displayed significant levels of phylogenetic signal. However, most studies also yielded a $K_{\mathrm{mult}}<1$, implying that the data contained less phylogenetic signal than expected under Brownian motion. Many empirists had interpreted this result as revealing “significant, but weak” phylogenetic signal. But an alternative possibility, noted by Adams and Collyer (2019), was that phylogenetic signal may be concentrated in one or a few trait dimensions. They argued that in such cases, $K_{\mathrm{mult}}$ would reveal significant phylogenetic signal (as compared with a random association of phenotypic data to the tips of the phylogeny), but less phylogenetic signal than expected if all trait dimensions evolved under Brownian motion. Computer simulations confirmed this supposition (Adams and Collyer, 2019), thereby revealing that $K_{\mathrm{mult}}$ was capable of identifying the presence of phylogenetic signal in multivariate data, but not whether this signal was concentrated in one or a few dimensions.

To determine whether different trait dimensions display differing levels of phylogenetic signal, a first step in a multivariate setting may thus be to extract features (linear combinations) with maximal phylogenetic signal. With a similar aim, Collyer and Adams (2021) presented an approach that aligns multivariate phenotypic data with phylogenetic signal, but this approach does not directly maximize any of the standard measures of phylogenetic signal. Here, we present a new method to explore phylogenetic signal in multivariate phenotypes. The key idea is to decompose the data into linear combinations with maximal (or minimal) phylogenetic signal, as measured by Blomberg’s K. Similar to PCs, the loading vectors of these “phylogenetic components” or “K-components” can be biologically interpreted, and scatterplots of the scores can be used as a low-dimensional ordination of the data that maximally (or minimally) preserves the phylogenetic signal. We refer to this approach as K-component analysis (KCA).

## Extending Blomberg’s K

Blomberg’s K is a common statistic to quantify phylogenetic signal (Blomberg et al., 2003). It is based on the ratio of the sum of squares of the observed tip data (${\text{SS}}_{\mathrm{obs}}$) divided by the sum of squares of these data after correcting for their expected dependences under a given model of evolution, usually a Brownian motion model (${\text{SS}}_{\mathrm{cor}}$). This latter quantity is computed by phylogenetic generalized least squares (PGLS) using a variance–covariance matrix derived from the candidate tree. The notion underlying the approach is that if the phenotypic distances between species resemble the phylogenetic distances (and in this sense show a strong phylogenetic signal), correcting for phylogenetic relatedness removes a large part of the interspecific trait variance, which leads to a large K statistic. In the case of no phylogenetic signal, this correction removes little variance, leading to a small K. The ratio is a dimensionless quantity but it is influenced by the structure of the tree. To make it compareble across studies, it is usually divided by its expectation under the given evolutionary model:


$$K=\frac{{\text{SS}}_{\text{obs}}/{\text{SS}}_{\text{cor}}}{E\,\left({\mathrm{SS}}_{\mathrm{obs}}/{\mathrm{SS}}_{\mathrm{cor}}\right)}.$$ (1)

Standardized this way, K<1 implies that relatives resemble each other less than expected under the given evolutionary model along the candidate tree, for example, due to adaptive evolution (homoplasy). K>1 implies that close relatives are more similar than expected.

Let the n×1 vector x contain a measured trait for n species, and write Ω for the n×n phylogenetic variance–covariance matrix. Under a Brownian motion model of evolution, the diagonal elements of Ω contain the phylogenetic distances from each tip to the root of the phylogeny, and the off-diagonal elements contain the phylogenetic distances from the root of the tree to the most recent common ancestor for each pair of species (this matrix of the Brownian motion model is usually denoted by **C**; Martins and Hansen, 1997; Rohlf, 2001). In principle, Ω can also be the expected covariance matrix under a different, non-Brownian model of evolution, or the phylogenetic covariances can be down-scaled by Pagel’s λ if appropriate (Pagel, 1999, see also the Supplementary Materials). The residuals of a variable from the phylogenetic mean are then equal to


$$x_{*}=x-1\,{\left[{\left(1^{T}\,\Omega^{-1}\,1\right)}^{-1}\,1^{T}\,\Omega^{-1}\,x\right]}^{T},$$ (2)

and


$${\mathrm{SS}}_{\mathrm{cor}}=x_{*}^{T}\,\Omega^{-1}\,x_{*}.$$ (3)

The superscript${\;}^{T}$ represents the transpose operator.

Blomberg et al. (2003) proposed two different ways of calculating the observed sum of squares (${\mathrm{SS}}_{\mathrm{obs}}$): as deviations from the phylogenetically corrected mean, $x_{*}^{T}\,x_{*}$, and as the ordinary sum of squares around the mean, ${\left(x-\bar{x}\right)}^{T}\,\left(x-\bar{x}\right)$. Even though most authors have used the first choice for calculating Blomberg’s K, it seems more consistent to us to use the ordinary least squares mean for calculating the observed SS when contrasting them with the PGLS sum of squares. The ratio of OLS to PGLS sum of squares also simplifies the permutation approach (see below) and the likelihood function (as shown in the Supplementary Materials). Accordingly, we will use the OLS sum of squares in the following notation, but both options can be used for the presented approaches. Numerically, they are very similar for most applications, and they are identical for a balanced phylogenetic tree. To ease notation, we will write x for the OLS mean centered variable (implying that $x^{T}\,1=0$) and $x_{*}$ for the PGLS centered variable. Blomberg’s K is then given by:


$$\begin{array}{ccc} K & = & \frac{{SS}_{obs}/{SS}_{cor}}{E({SS}_{obs}/{SS}_{cor})}\\ & = & x^{T}x/x_{*}^{T}\Omega^{-1}x_{*}/\epsilon , \end{array}$$ (4)

where


$$\epsilon =\left(\mathrm{tr}\,\Omega -\left(1^{T}\,\Omega^{-1}\,1\right)\,n^{-1}\right)\,{\left(n-1\right)}^{-1}$$ (5)

standardizes the observed ${\mathrm{SS}}_{\mathrm{obs}}/{\mathrm{SS}}_{\mathrm{cor}}$ ratio by the expected ratio under a Brownian motion model (Blomberg et al., 2003; Adams, 2014).

For multivariate data, consider the OLS meancentered n×p data matrix X and the matrixof residuals from the phylogenetic mean vector$X_{*}=X-1\,{\left[{\left(1^{T}\,\Omega^{-1}\,1\right)}^{-1}\,1^{T}\,\Omega^{-1}\,X\right]}^{T}$. The univariate ${\mathrm{SS}}_{\mathrm{obs}}$ then translates into a p×p matrix of sum of squares and cross-products (${\text{SSCP}}_{\mathrm{obs}}$), $X^{T}\,X$, and an ${\text{SSCP}}_{\mathrm{cor}}$ matrix of the phylogenetically corrected data, $X_{*}^{T}\,\Omega^{-1}\,X_{*}.$ As a multivariate extension of K, Adams, (2014) proposed the ratio of the traces of these two matrices:


$$\begin{array}{ccc} K_{mult} & = & \frac{tr({SSCP}_{obs})}{tr({SSCP}_{cor})}\epsilon^{-1}\\ & = & \frac{tr(X^{T}X)}{tr(X_{*}^{T}\Omega^{-1}X_{*})}\epsilon^{-1}. \end{array}$$ (6)

Following Blomberg et al. (2003), Adams (2014) used residuals from the phylogenetic mean to calculate ${\mathrm{SSCP}}_{\mathrm{obs}}$, but for consistency we use the OLS estimate for ${\mathrm{SSCP}}_{\mathrm{obs}}$ here (For $K_{\mathrm{mult}}$, the use of OLS or PGLS results in slightly different but highly rank-correlated values; see Supplementary Material.) Moreover, Adams expressed $K_{\mathrm{mult}}$ in terms of Euclidean distances, but the formulation presented here is numerically identical with the original. $K_{\mathrm{mult}}$ can be a useful summary statistic if all variables show a similar magnitude of phylogenetic signal. However, if the measured variables comprise features with different phylogenetic signals, it can be important to disentangle these signals by first identifying the phenotypic features that carry these diverse signals.

For such an exploratory approach, construct the p×p phylogenetic signal matrix


$$K={\mathrm{SSCP}}_{\mathrm{cor}}^{-1}\,{\mathrm{SSCP}}_{\mathrm{obs}}\,E\,{\left({\mathrm{SSCP}}_{\mathrm{cor}}^{-1}\,{\mathrm{SSCP}}_{\mathrm{obs}}\right)}^{-1}.$$

Here, we assume that X is of full rank and ${\text{SSCP}}_{\mathrm{cor}}$ is invertible (but see “Numerical Properties” section). Under the simplest model, independent Brownian motion of every variable, the expectation is just an identity matrix multiplied by ϵ (Equation (5)):


$$E\,\left({\mathrm{SSCP}}_{\mathrm{cor}}^{-1}\,{\mathrm{SSCP}}_{\mathrm{obs}}\right)=\epsilon \,I.$$

In this simple case, multiplying with the inverse of ϵ⁢I is the same as element-wise division by ϵ. Hence, we can write


$$\begin{array}{ccc} K & = & {SSCP}_{cor}^{-1}\;{SSCP}_{obs}\;\epsilon^{-1}\\ & = & (X_{*}^{T}\Omega^{-1}X_{*}{)}^{-1}X^{T}X\;\epsilon^{-1}. \end{array}$$ (7)

Decompose K into


$$K=E\,\Delta \,E^{-1},$$ (8)

where E is a matrix of eigenvectors and Δ a diagonal matrix of eigenvalues. Technically, this is a relative eigenanalysis of ${\mathrm{SSCP}}_{\mathrm{obs}}$ with respect to ${\mathrm{SSCP}}_{\mathrm{cor}}$ (Flury, 1985; Bookstein and Mitteroecker, 2014; Le Maitre and Mitteroecker, 2019). The first eigenvector, $e_{1}$, corresponds to the linear combination, or direction in data space, with maximal K, which is equal to $\delta_{1}$. We term this vector the first phylogenetic component, or more specifically, the first K-component . The second eigenvector, $e_{2}$, has the second highest K, and so forth. The elements of these eigenvectors can be interpreted as the loadings of the measured variables on the linear combinations with maximal phylogenetic signal. Phrased differently, these are the linear combinations that are most affected by phylogenetic correction and in this sense carry the highest phylogenetic signal.

Scores along these dimensions can be computed for the observed data $s_{i}={\mathrm{Xe}}_{i}$ as well as for the phylogenetically corrected data $t_{i}=\Omega^{-1/2}\,X_{*}\,e_{i}$, where


$$\begin{array}{ccc} K_{i} & = & \frac{s_{i}^{T}s_{i}}{t_{i}^{T}t_{i}}\\ & = & \frac{e_{i}^{T}X^{T}{Xe}_{i}}{e_{i}^{T}X_{*}^{T}\Omega^{-1}X_{*}e_{i}}\\ & = & \delta_{i} \end{array}$$ (9)

and $K_{1}\geq K_{2}\geq \ldots \geq K_{p}.$ As K usually is not symmetric, the eigenvectors $e_{i}$ usually are not orthogonal, but the scores are mutually uncorrelated both for the observed data and for the corrected data: $\mathrm{cor}\,\left(s_{i},s_{j}\right)=\mathrm{cor}\,\left(t_{i},t_{j}\right)=0$ for all i≠j.

Scatterplots of the scores serve as ordinations of the data that maximize or minimize phylogenetic signal. Specifically, a plot of $s_{1}$ versus $s_{2}$ represents variation with the strongest phylogenetic signal, whereas a plot of $s_{p}$ versus $s_{p-1}$ represents variation with the least phylogenetic signal. Accordingly, $t_{1}$ and $t_{2}$ represent variation in the components with strongest phylogenetic *after* that signal has been removed. Contrasting the plots of $s_{1}$ versus $s_{2}$ and of $t_{1}$ versus $t_{2}$ may be useful to gauge the effect of phylogenetic correction for further analyses. Similar to phylogenetically aligned component analysis (PACA; Collyer and Adams, 2021), these scatterplots of the KCA present phenotypic variation in relation to phylogenetic signal, though our approach specifically maximizes Blomberg’s K.

## Numerical Properties

As is typical for multivariate methods that maximize a certain statistic, the maximal variance ratios, $K_{i}$, tend to increase with p/n (e.g., Mitteroecker and Bookstein, 2011; Bookstein, 2017). Intuitively, this is because with increasing p/n random noise accumulates and may accidentally lead to large variance ratios. But the corresponding linear combinations typically are biologically meaningless and have unreliable out-of-sample properties, similar to a strongly overfitted linear model. If **X** is not of full rank, for example, because p≥n, the matrix K cannot be computed at all because ${\mathrm{SSCP}}_{\mathrm{cor}}$ is not invertible. In our experience, a reliable interpretation requires that the number of species exceeds the number of variables by at least 4–5 times. In geometric morphometrics and other highly multivariate fields, where the number of variables often exceeds the number of cases, this may require dimension reduction or matrix regularization.

One simple way of dimension reduction is to decompose $X^{T}\,X=V\,\Theta \,V^{T}$, so that $Z={\mathrm{XV}}_{1\,\ldots \,k}$ are the first k PC scores of X. The matrix K in Equation (7) and its eigenvectors E can be computed based on Z instead of X. For interpreting the loadings of the K-components in terms of the original variables, the eigenvectors E then need to be transformed back as $V_{1\,\ldots \,k}\,E$. The scores can be computed as ${\mathrm{XV}}_{1\,\ldots \,k}\,E=\mathrm{ZE}.$ If k is set to the rank of $X_{*}$, this approach is equal to using the Moore–Penrose pseudoinverse of ${\mathrm{SSCP}}_{\mathrm{cor}}$ instead of the regular matrix inverse in Equation (7). In most applications, however, k≪rank⁢(X). PCA proved to be useful in morphometrics because of the highly correlated measurements and the possibility to inspect the components used for further analysis (e.g., Mitteroecker and Schaefer, 2022), but dimension reduction can modify the phylogenetic signal to some degree. For multivariate data with a strong phylogenetic structure, the first PCs likely contrast the taxa on either side of the deepest nodes, whereas more recent divergence might be reflected in later PCs. Discarding these later PCs can thus inflate the existing phylogenetic signal (Polly et al., 2013). Similarly, omitting PCs with little or no phylogenetic signal reduces the anisotropy of the signal (see below). Choosing the number of PCs for phylogenetic analysis thus requires careful consideration and repeating the analysis with a range of PCs. In our experience, a large part of the phylogenetic signal is captured by the first few PCs. Further increasing the number of PCs for calculating K leads to a range of PCs for which the eigenvectors are relatively consistent. Above this range, the results become noisy and uninterpretable, indicating too high a p/n-ratio. Depending on the data structure and biological context, other methods of dimension reduction, such as PACA (Collyer and Adams, 2021), intrinsic relative warps or partial warps (Bookstein, 2015; Mitteroecker et al., 2020), can be slightly more effective than ordinary PCA, but we found that after the first few components captured the phylogenetic signal, they all performed similarly (see “Empirical Examples” section). Clearly, phylogenetic PCA (Revell, 2009; Polly et al., 2013) should not be used for dimension reduction in this context as it aims to reduce rather than concentrate phylogenetic signal.

Because the eigenvalues of K generally become larger and more anisotropic with p/n (see Supplementary Table S1), the absolute values of $K_{i}$ should not be interpreted. Therefore, the correction by e can also be omitted. The main aim of our approach is to identify linear combinations of the measured variables that carry maximal or minimal phylogenetic signal. Hence, most interesting will be the eigenvectors of K (the K-components), which contain the loadings of the linear combinations. Nonetheless, a “scree plot” of the eigenvalues can be helpful to gauge which and how many K-components carry most of the phylogenetic signal.

## Summary Metrics

As a scalar summary metric of multivariate phylogenetic signal, Adams (2014) proposed $K_{\mathrm{mult}}$, the ratio of the traces of the ${\text{SSCP}}_{\mathrm{obs}}$ and ${\text{SSCP}}_{\mathrm{cor}}$ matrices (Equation (6)). An alternative metric, $K_{A}$, is given by the arithmetic mean of the eigenvalues of K, which is equal to the trace of K divided by p:


$$K_{A}=\frac{1}{p}\,\sum_{i=1}^{p}\delta_{i}=\frac{1}{p}\,\mathrm{tr}\,\left(K\right).$$ (10)

The product of the eigenvalues is equal to the ratio of the generalized variances of the observed data and the phylogenetically corrected data:


$$\prod \delta_{i}=\frac{\det\,\left(X^{T}\,X\right)}{\det\,\left(X_{*}^{T}\,\Omega^{-1}\,X_{*}\right)}=\det\,\left(K\right).$$ (11)

This gives rise to another metric, the geometric mean of the eigenvalues of K:


$$K_{G}={\left(\prod_{i=1}^{p}\delta_{i}\right)}^{1/p}=\det\,{\left(K\right)}^{1/p}.$$

Under the hypothesis of equal phylogenetic signal for all dimensions, ${\mathrm{SSCP}}_{\mathrm{obs}}=k\,{\mathrm{SSCP}}_{\mathrm{cor}}$, the two SSCP matrices are proportional and $K_{\mathrm{mult}}=K_{A}=K_{G}=k$ . In this situation, all 3 indices lead to the same estimate of the “global” phylogenetic signal. In the presence of multivariate normal noise, the maximum likelihood estimate is


$$\hat{k}=\mathrm{tr}\,\left(K\right)/p=K_{A}$$ (12)

(see Mardia et al., 1979; Bookstein and Mitteroecker, 2014). However, the 3 metrics differ when heterogenous phylogenetic signal is present across trait dimensions and $K_{A}>K_{G}$. The ratio $K_{A}/K_{G}$ thus serves as a measure of the heterogeneity of phylogenetic signal across trait dimensions (see below). In contrast to $K_{A}$ and $K_{G}$, the calculation of $K_{\mathrm{mult}}$ does not require dimension reduction as it does not involve a matrix inverse.

Like the univariate K, the eigenvalues of K are affine invariant (Bookstein and Mitteroecker, 2014). That is, they remain unchanged when rescaling the variables separately or jointly (linear scaling or shearing of the data space, respectively). Thus, $K_{A}$ and $K_{G}$ are also affine invariant, which includes rotation invariance. By contrast, $K_{\mathrm{mult}}$ is not affine invariant, only rotation invariant (Adams and Collyer, 2018a), which implies that the statistics are the same whether computed from the original variables or from an orthogonal rotation of them (e.g., the full set of PCs). For geometric morphometric data, this further means that the orientation of the Procrustes-aligned landmark configurations does not influence the statistics. The affine invariance of $K_{A}$ and $K_{G}$ additionally implies that the variables do not need to have the same units, nor do the variables need to be geometrically independent. This can be important in traditional morphometrics and other fields where the variables have different units. Moreover, when based on the leading PCs of the variables, the eigenvalues of K are also approximately invariant to changes in the redundancy of variables. This can be important in geometric morphometrics, where the number and spacing of landmarks and especially of semilandmarks often is arbitrary (Huttegger and Mitteroecker, 2011; Mitteroecker and Schaefer, 2022). For these reasons, $K_{A}$ and $K_{G}$ tend to display greater statistical power for recognizing phylogenetic signal than $K_{\mathrm{mult}}$ (see below).

## Statistical Inference

Similar to other multivariate methods, significance tests of phylogenetic signal for single K-components are inappropriate because the K-values are maximized and depend on p/n. Due to this property, bootstrap distributions of K-values are also biased. Bootstrap samples contain only a subset of the cases from the original sample, and the effective p/n-ratio thus is larger in the bootstrap samples than in the original sample. Instead, the eigenvalues can be compared against a permutation distribution. Under the null hypothesis of phylogenetic independence (i.e., a random association of phenotypic values to the phylogeny; Blomberg et al., 2003), phenotypic residuals can be randomly permuted while leaving the phylogeny unchanged, resulting in a distribution that preserves both first- and second-moment exchangeability (sensu Commenges, 2003; Adams and Collyer, 2018b, 2022).

To test the multivariate null hypothesis of complete phylogenetic independence, any of the 3 summary metrics of phylogenetic signal described above, $K_{m\,u\,l\,t}$, $K_{A}$, or $K_{G}$ , can be compared against its permutation distribution. Computing ${\text{SSCP}}_{\mathrm{obs}}$ from the OLS centered residuals (as suggested in Equation (7)) simplifies the permutation tests because ${\text{SSCP}}_{\mathrm{obs}}$ is invariant to the permutation of **X** and does not need to be recomputed. The permutation test of $K_{m\,u\,l\,t}$ then reduces to the permutation of tr(${\text{SSCP}}_{\mathrm{cor}}$) and that of $K_{G}$ to det(${\text{SSCP}}_{\mathrm{cor}}$). After rejecting the null hypothesis of complete phylogenetic independence, the linear combinations of the variables that carry this signal can be explored by the leading K-components (the first eigenvectors of **K**).

For complex anatomical structures, phylogenetic signal is likely to vary across traits because adaptation, genetic drift, and developmental constraints differentially affect different traits. Nonetheless, the $H_{0}$ of completely uniform phylogenetic signal can also be tested formally. This $H_{0}$ implies that ${\mathrm{SSCP}}_{\mathrm{obs}}=k\,{\mathrm{SSCP}}_{\mathrm{cor}}$, that is, the 2 matrices are proportional, and that all eigenvalues of **K** are equal:


$$H_{0}:\delta_{1}=\delta_{2}=\ldots =\delta_{p}.$$ (13)

Note that the $H_{0}$ can also reflect Ω=d⁢I, which corresponds to a star phylogeny and a Pagel’s λ of 0. The ratio of arithmetic to geometric means of eigenvalues, $K_{A}/K_{G}$, is 1 under the $H_{0}$ and increases with increasing anisotropy of phylogenetic signal. This ratio thus serves as a statistic to test the $H_{0}$ of completely uniform signal (cf. Mardia et al., 1979; Le Maitre and Mitteroecker, 2019). As $K_{A}/K_{G}$ is invariant to k, its permutation distribution can also be inferred from the permutation of cases against the phylogeny. However, in a series of simulation studies and empirical examples (see below and Supplementary Materials), we found that this test requires n≫p and that it can be incompatible with dimension reduction. If reducing data to the dimensions that collectively contain the phylogenetic signal, the anisotropy of the signal is necessarily lower as if dimensions with little or no phylogenetic signal were included. Conversely, if all variables indeed had equal phylogenetic signal, PCA concentrates part of this signal in the leading dimensions. Assessing only these dimensions can incorrectly suggest the presence of “significant” anisotropy of the signal. For these reasons, we recommend to use this test of uniform phylogenetic signal only for small numbers of measured traits and large sample sizes, without the use of dimension reduction.

Instead of the actual test statistics, Collyer et al. (2015, 2022) suggested expressing these statistics as Z-scores of their (normalized) permutation distribution:


$$Z=\frac{\theta_{o\,b\,s}-\mu_{\theta }}{\sigma_{\theta }},$$ (14)

where $\theta_{o\,b\,s}$ is the test statistic for the observed data (e.g., $K_{G}$), and μ and σ are the mean and standard deviation of the normalized permutation distribution, respectively. These scores can be used to compare the ability to detect phylogenetic signal (i.e., the relative statistical power) across the 3 statistics, $K_{m\,u\,l\,t}$, $K_{A}$, and $K_{G}$.

As an alternative to hypothesis tests, one can use a cross-validation approach to gauge the efficacy of the K-components to represent phylogenetic signal. For instance, the fraction of taxa correctly classified into larger clades based on the first few K-components can be computed from leave-one-out or k-fold cross-validation. This way, also the optimal number PCs for calculating K can be evaluated.

## Simulation Examples

We performed a series of stochastic sampling experiments to demonstrate the ability of these approaches to detect patterns of multivariate phylogenetic signal under varying conditions. Our simulation design was similar to that of Adams and Collyer (2019), where phenotypic data were simulated such that the various trait dimensions contained differing levels of phylogenetic signal.

For the first simulation, we generated a pure-birth phylogeny for n=60 species, and for all species we simulated multivariate phenotypes containing p=5 trait dimensions under a Brownian motion model of evolution. This procedure was equivalent to drawing n=60 phenotypic values from a multivariate normal distribution as: $Y_{B\,M}∼𝒩\,\left(0,\Omega_{n}\right)$, where $\Omega_{n}$ was the phylogenetic covariance matrix. Next, we generated a second multivariate dataset independent of the phylogeny (i.e., independent random noise), by drawing phenotypes containing P=5 trait dimensions from a multivariate normal distribution as: $Y_{R}∼𝒩\,\left(0,I_{n}\right)$. From these 2 initial datasets, we generated 4 datasets containing varying levels of phylogenetic signal:

1) $Y_{H}=Y_{B\,M}$ (high phylogenetic signal). Here, all 5 traits contained phylogenetic signal at levels expected under Brownian motion.

2) $Y_{M}=\left(Y_{B\,M}+Y_{R}\right)/2$ (medium phylogenetic signal). Here, all 5 traits contained similar levels of phylogenetic signal, but at half of what was expected under Brownian motion.

3) $Y_{L}=\left(Y_{B\,M}+2\,Y_{R}\right)/3$ (low phylogenetic signal). Here, all 5 traits contained similar levels of phylogenetic signal, but at a third of what was expected under Brownian motion.

4) $Y_{C}=\left[\begin{array}{cc} Y_{B\,M}\left[1:2\right] & Y_{R}\left[3:5\right] \end{array}\right]$ (concentrated phylogenetic signal). Here, the first 2 traits contained phylogenetic signal as expected under Brownian motion, while the remaining 3 traits contained random noise.

For each dataset we calculated the phylogenetic signal matrix, **K** (Equation (7)), and obtained the set of eigenvalues, $\delta_{1}\,\ldots \,\delta_{5}$. We then calculated 95% intervals of the permutation distributions for these eigenvalues based on 1000 random permutations. The expectation was that the eigenvalues from our procedure would reflect the magnitude of phylogenetic signal across simulation conditions, that is, $\delta_{H}>\delta_{M}>\delta_{L}$. Additionally, we expected that the distribution of eigenvalues from the dataset containing concentrated phylogenetic signal ($Y_{C}$) would differ from the distribution of eigenvalues from the remaining datasets. As a measure of the anisotropy of phylogenetic signal, we expect $K_{A}/K_{G}$ to be similar in datasets 1–3 and higher in dataset 4.

The second simulation was identical in design to the first simulation, except that the procedure was repeated 1000 times (i.e., 1000 datasets were simulated as above). From all these datasets we claculated the summary statistics $K_{m\,u\,l\,t}$, $K_{A}$, $K_{G}$ (Equation (6), Equation (10), Equation (11)) and their corresponding Z-scores (Equation (14)). The statistical power of the 3 statistics was estimated as the fraction of significant results at P<0.05.

Finally, as high-dimensional datasets often contain more variables than observations (P>n), we repeated the above simulations with P=100 and n=60 and a dimension reduction step. Details are found in theSupplementary Materials. All simulations were performed in R, using routines written by the authors (available in the Supplementary Materials).

### Simulation Results

**Figure 1:**
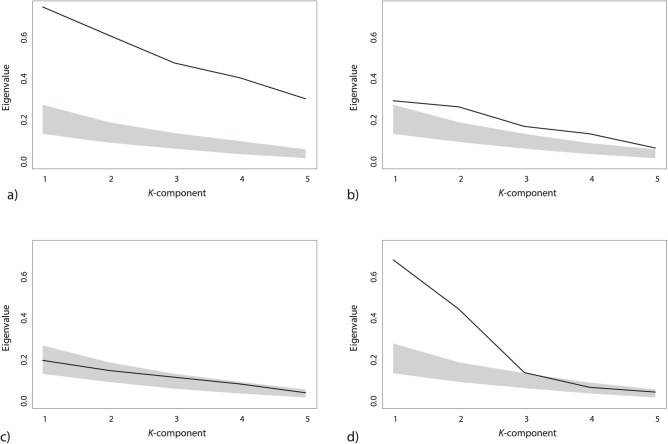
Results from a single simulation run of multivariate data (p=5 trait dimensions) for 60 species related by a pure-birth phylogeny. Each panel displays the observed eigenvalues obtained from the multivariate phylogenetic signal matrix, **K**, as well as the 95% intervals of the permutation distributions. (a) Dataset containing uniformly high phylogenetic signal, (b) medium phylogenetic signal, (c) low phylogenetic signal, and (d) phylogenetic signal concentrated in only 2 dimensions.

The first simulation demonstrated that the eigenvalues of **K** correctly captured known levels of phylogenetic signal in the data. The eigenvalues for the dataset containing high levels of phylogenetic signal were larger (Fig. 1A) than those for the datasets containing medium (Fig. 1B) and low levels of phylogenetic signal (Fig. 1C), respectively. Additionally, as levels of phylogenetic signal in the data decreased, the observed eigenvalues approached (or overlapped) with the 95% confidence intervals of the permutation distribution. This implies that statistical tests based on these eigenvalues would follow in rank-order with known input levels of phylogenetic signal, as expected. Finally, for dataset 4, where the phylogenetic signal was concentrated in 2 trait dimensions, the first 2 eigenvalues were considerably larger than the remaining eigenvalues, which fell within the permutation distribution (Fig. 1D). Accordingly, $K_{A}/K_{G}$ was much larger in dataset4 ($K_{A}/K_{G}=2.355$) than in the first 3 datasets (1.216, 1.423, 1.381). Note that $K_{A}/K_{G}=1$ for exactly equal eigenvalues and increases with increasing heterogeneity of eigenvalues. The permutation test of $K_{A}/K_{G}$ was only significant for dataset 4 (P<0.001).

**Figure 2:**
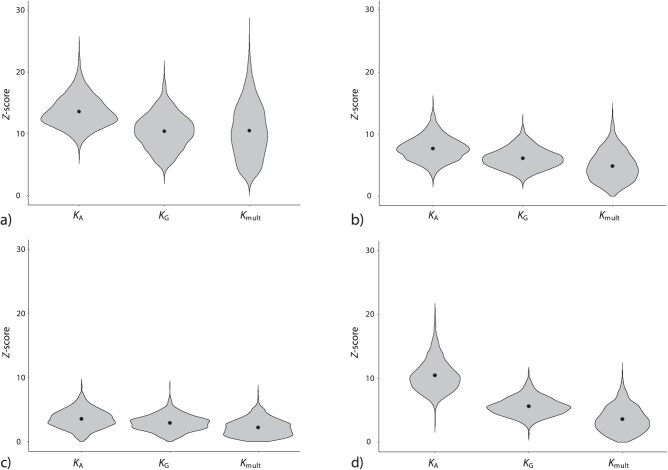
Summary test measures for 1000 simulated datasets containing differing levels of phylogenetic signal. (a) Violin plots of the Z-scores for datasets containing high phylogenetic signal, (b) medium phylogenetic signal, (c) low phylogenetic signal, (d) and phylogenetic signal concentrated in 2 dimensions.

Results from the second simulation revealed that Z-scores for 3 summary test measures corresponded well with input levels of phylogenetic signal, with the highest Z-scores obtained for datasets with high levels of phylogenetic signal, and the lowest Z-scores obtained for datasets with low levels of phylogenetic signal (Fig. 2A–C). For all 4 simulations, $K_{A}$ tended to have the highest Z-scores and $K_{m\,u\,l\,t}$ the lowest, reflecting the maximum likelihood property of $K_{A}$ as the scaling factor between ${\text{SSCP}}_{\mathrm{obs}}$ and ${\text{SSCP}}_{\mathrm{cor}}$ under $H_{0}$ (see Equation (12)). Moreover, the permutation distribution for $K_{m\,u\,l\,t}$ was more leptokurtic and right-skewed than for the other 2 statistics.

The difference between statistics was particularly prominent when phylogenetic signal was concentrated in 2 dimensions (dataset 4; Fig. 2D). Here, the distribution of Z-scores for $K_{m\,u\,l\,t}$ was similar to its distribution for uniformly low phylogenetic signal, reflecting the difficulty of interpreting “significant, but weak” phylogenetic signal from $K_{m\,u\,l\,t}$. Conversely, for $K_{A}$ the distribution was in-between that of uniformly high and medium phylogenetic signal. These trends were consistent with the summaries of statistical power, showing that $K_{A}$ and $K_{G}$ attained higher power as compared with $K_{m\,u\,l\,t}$ (Table 1), particularly for uniformly low and concentrated phylogenetic signal (datasets 3 and 4).

**Table 1:** Statistical power of the 3 summary statistics for the 1000 simulated datasets containing various levels of phylogenetic signal.

Phy. Signal	$K_{A}$	$K_{G}$	$K_{m\,u\,l\,t}$
High	1.000	1.000	1.000
Medium	1.000	1.000	0.926
Low	0.913	0.847	0.570
Concentrated	1.000	0.998	0.808

Finally, the simulations using high-dimensional data (P>n) and dimension reduction obtained results that mirrored those presented above. The eigenvalues of **K** correctly captured known levels of phylogenetic signal in the data. Likewise, when phylogenetic signal was concentrated, $K_{A}$ and $K_{G}$ were better capable of identifying this signal than was $K_{m\,u\,l\,t}$. But in contrast to the simulations above, $K_{A}/K_{G}$ was similarly large and significant for all four datasets, showing the inappropriateness of this test for p>n (see Supplementary Materials for details).

## Empirical Example 1: Crocodyliform Skull Shape

**Figure 3:**
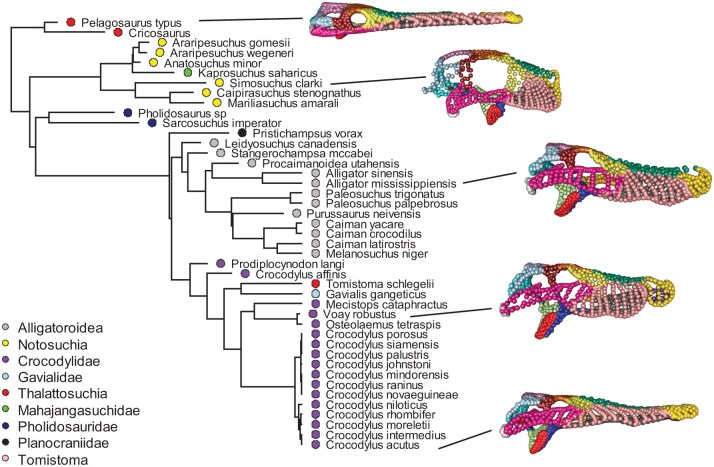
Time-calibrated phylogeny of fossil and extant crocodyliforms. Major clades (following Felice et al., 2021) along with landmark data for several representative specimens are displayed (color scheme of landmarks designate distinct cranial regions, as based on the original publication).

To illustrate the utility of our approach on empirical data we present 2 worked examples. The first one investigates the evolution of skull morphology in 43 species of modern and fossil crocodyliforms (24 extant taxa and 19 fossil lineages; Fig. 3). The data were originally from Felice et al. (2021) and are available at github.com/rnfelice/Croc_Skulls. On 3D scans of 428 skulls, a total of 1364 3D landmarks and semilandmarks were placed on the right side and along the midline of each scan. Semilandmarks were slid in order to minimize the bending energy of each configuration to the mean shape (Bookstein, 1997; Gunz and Mitteroecker, 2013). All configurations were then superimposed by Generalized Procrustes Analysis(Rohlf and Slice, 1990). Finally, specimens were matched to several time-calibrated phylogenies for subsequent macroevolutionary analyses (for details see Felice et al., 2021).

The original study evaluated morphological evolution in the group using an extensive array of phylogenetic comparative analyses and modeling approaches. Among those analyses, an evaluation of phylogenetic signal obtained a value of $K_{m\,u\,l\,t}=0.06$ (for the phylogeny in Figure 3), and permutation tests revealed that this value was significantly greater than under the hypothesis of complete phylogenetic independence. Felice et al. (2021) interpreted this result as “significant, but weak” phylogenetic signal. However, because such patterns can also be obtained when phylogenetic signal is concentrated in one or a few trait dimensions, it is of interest to interrogate the data using KCA.

As the number of variables for this example (P=3873) was orders of magnitude greater than the number of species (n=43), dimension reduction was required. For this we used the first 20 PCs, which collectively explained nearly 97% of the total variation in shape. Additionally, and for comparison, we also used the first 20 PACs (Collyer and Adams, 2021). We then evaluated patterns of phylogenetic signal using the procedures described above, with 100,000 permutations used for the significance tests. All statistical analyses were performed in R, using the packages RRPP 2.0.0 (Adams and Collyer, 2018b; Collyer and Adams, 2024), geomorph 4.0.5 (Baken et al., 2021), and routines written by the authors (available as functions in newer versions of geomorph).

### Results

Our reanalysis of the data using 20 PCs revealed significant phylogenetic signal for all metrics ($K_{m\,u\,l\,t}=0.069$; $P_{K_{m\,u\,l\,t}}<0.001$; $K_{A}=0.264$; $P_{K_{A}}<0.001$; $K_{G}=0.074$; $P_{K_{G}}<0.0001$). Consistent with our simulation studies, Z-scores for the 3 metrics indicated the strongest signal for $K_{A}$ and $K_{G}$ ($Z_{K_{m\,u\,l\,t}}=3.091$; $Z_{K_{A}}=5.454$; $Z_{K_{G}}=6.046$).

**Figure 4:**
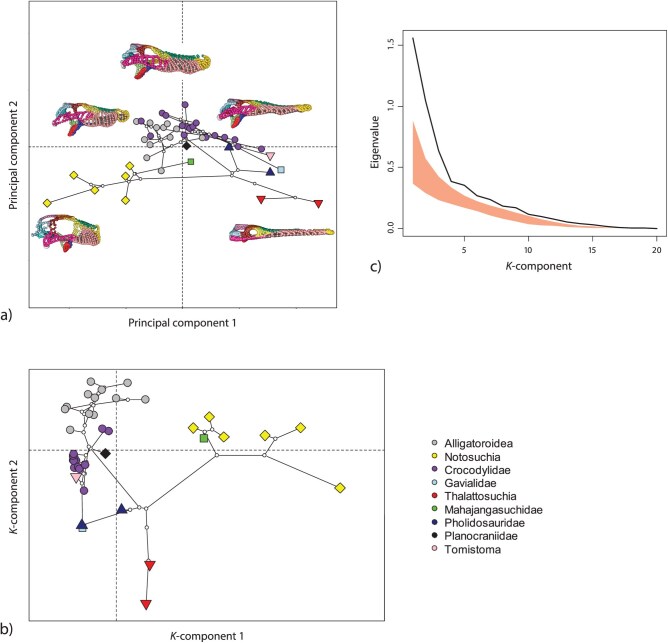
(a) First two PCs of crocodyliform skull shape. The landmark configurations of representative specimens are displayed in their approximate position in PC space. The species’ phylogeny is projected into this space to facilitate interpretation (ancestral shapes were estimated by PGLS and a Brownian motion model of evolution). (b) First two K-components of crocodyliform skull shape. These are the linear combinations of the shape coordinates with maximal phylogenetic signal. (c) Eigenvalues of **K** for the crocodyliform data (black line) representing the relative strength of phylogenetic signal for each K-component. The red polygon represents the 95% interval from 1000 random permutations. A colour version of this figure appears in the online version of this article.

The first PCs of the shape coordinates (69% of total variance) already revealed clusters of species by clade (Fig. 4a), but the phylogenetic signal was much clearer in the first two K-components (Fig. 4b). These are the scores with maximal K-value ($s_{i}$ in Equation (9)). Visualizing the eigenvalues of the **K** matrix indeed revealed the presence of concentrated phylogenetic signal, as the first several eigenvalues fell well outside of the 95% confidence limits of the permutation distribution (Fig. 4b,c). There was a precipitous decline across eigenvalues, with the first eigenvalue displaying 50% greater phylogenetic signal than the second, and a similar drop off to the third. Further, the higher eigenvalues of this distribution were much more similar to what was expected via permutation, corroborating that the phylogenetic signal in the dataset was largely concentrated in the first K-components. Results using the first 20 PACs dimensions were very similar (see Supplementary Materials). Thus, our re-analysis of the crocodyliform dataset suggests the presence of strong and concentrated phylogenetic signal in skull shape, rather than a “significant, but weak” signal.

## Empirical Example 2: Papionin Cranial Shape

**Figure 5:**
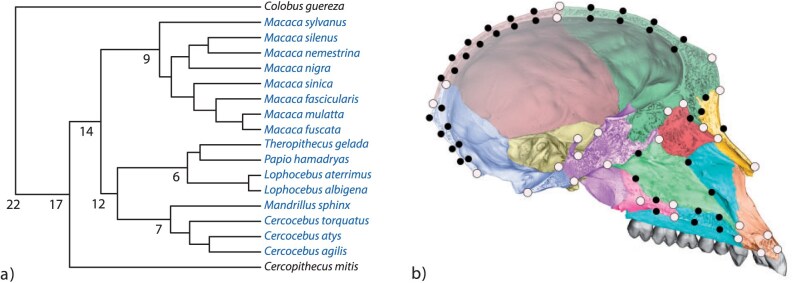
(a) Molecular phylogeny of the 16 papionin taxa (blue) and the 2 outgroup Old World monkey taxa (black), based on several mitochondrial, Y-chromosomal, and autosomal markers (Arnold et al., 2010). Approximate divergence times are given in million years. (b) Landmark scheme shown for a *Macaca fuscata* specimen. The different cranial bones are shown in different colors. The 28 anatomical landmarks are shown in gray and the 42 sliding landmarks in black (modified from Grunstra et al., 2021). A colour version of this figure appears in the online version of this article.

Our second example explores phylogenetic signal in the cranial shape of papionins and provides a more in-depth inspection and interpretation of components. Our data are from Grunstra et al. (2021) and represent the average midsagittal cranial shape of 16 different papionin species as well as 2 nonpapionin Old World monkey taxa, *Cercopithecus mitis* and *Colobus guereza*, which belong to the sister taxon of the papionini (the cercopithecini) and the sister taxon to the cercopithecinae (the colobinae), respectively (Fig. 5a). Only adult females were sampled in order to minimize ontogenetic variation and sexual dimorphism. A total of 28 anatomical landmarks and 42 sliding landmarks were measured on 3D-computed tomography scans by Silvester Bartsch and Nicole Grunstra (Fig. 5b; landmark data are available at doi.org/10.5061/dryad.zkh189373). The semilandmarks were slid in order to minimize the bending energy of each configuration to the mean shape (Bookstein, 1997; Gunz and Mitteroecker, 2013), and then all the configurations were superimposed by Generalized Procrustes Analysis (Rohlf and Slice, 1990). KCA was based on the first 5 PCs, but the analyses were repeated with different numbers of PCs. One hundred thousand permutations were used for the significance tests, and the phylogeny was rescaled to unit depth (see Supplementary Material). Analyses were performed in Wolfram Mathematica 12.3 using functions written by Philipp Gunz and Philipp Mitteroecker. Phylomorphospace plots were computed using Mathematica code by David Polly (2022).

### Results

**Figure 6:**
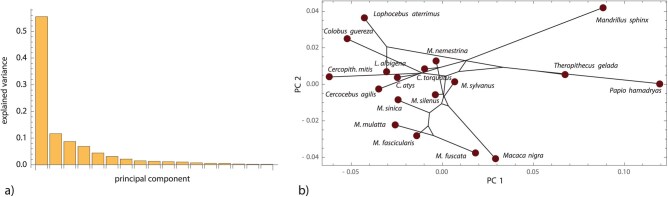
(a) Scree plot of the papionin cranial shape data, showing the fraction of variance for each PC. (b) The first two PC scores along with a phylogenetic tree projected in this PC space (ancestral shapes were estimated by PGLS and a Brownian motion model of evolution).

Despite the small sample, all 3 metrics revealed a significant phylogenetic signal in the data ($K_{m\,u\,l\,t}=0.166$; $P_{K_{m\,u\,l\,t}}=0.0279$; $K_{A}=0.493$; $P_{K_{A}}=0.0016$; $K_{G}=0.437$; $P_{K_{G}}=0.0002$). As in the simulation studies and the previous example, Z-scores for the 3 metrics indicated the strongest signal for $K_{A}$ and $K_{G}$ ($Z_{K_{m\,u\,l\,t}}=1.793$; $Z_{K_{A}}=2.868$; $Z_{K_{G}}=3.298$). Levels of overall phylogenetic signal were less than expected under a Brownian motion model of evolution (i.e., $K_{m\,u\,l\,t}<1$), which may indicate the presence of concentrated phylogenetic signal. Also, the first 2 PCs of the shape coordinates (accounting for 67% of total variance) did not represent phylogenetic relationships well (Fig. 6).

**Figure 7:**
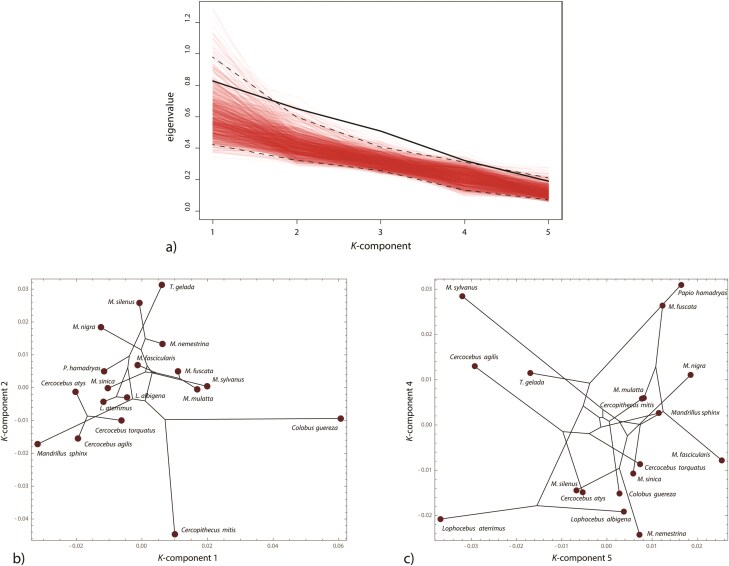
(a) Eigenvalues of the **K** matrix for the papionin data (thick black line), representing the relative strength of phylogenetic signal for every K-component. There are only 5 eigenvalues as the computation of **K** was based on the fist 5 PCs of the shape data only. The thin red lines represent the eigenvalue profiles of 1000 random permutations, and the dashed lines their 95% intervals. (b) Scores of the 18 taxa along the two K-components with maximal phylogenetic signal and (c) the two K-components with minimal phylogenetic signal. The phylogenetic structure (as shown by the projected tree) is much better represented by the first 2 components as compared with the last 2 components and also with the ordinary PCs in Figure 6. A colour version of this figure appears in the online version of this article.

The **K** matrix derived from the first 5 PCs (87% of total variance) had eigenvalues (K-values) of: 0.8007, 0.6463, 0.5074, 0.3211, and 0.1889. Hence, the first K-component showed approximately 4 times as much phylogenetic signal than the last component. The observed K-value profile fell at the limit of the permutation distribution (Fig. 7a), reflecting the significant overall phylogenetic signal, but the decline of the observed eigenvalues was comparable to that of the permutation distribution. Even though the first component showed the highest eigenvalue, the eigenvalues of the second and third components, not the first, deviated most clearly from the permutation distribution, which seems counter-intuitive at first glance. However, because of the 2 outgroups (deep branches for common ancestors) and 2 distinct clades within the phylogenetic tree, one would expect a high frequency of random permutations of taxa that result in the maintained separation of distantly-related taxa into different clades, by chance. Examination of the permutations that yielded the largest eigenvalues suggested this was the case. The second and third components likely were more associated with within-clade phylogenetic signal.

Scores along the K-components (Fig. 7b,c) showed that the phylogenetic relationships among the taxa were much more clearly represented by the first two components (the components with maximal K) as compared with the last 2 components (with minimal K) and also better than by the ordinary PCs in Figure 6b. Note that these are the scores for the observed data ($s_{i}$ in Equation (9)). The first pair of scores for the phylogenetically corrected data ($t_{i}$) showed no phylogenetic structure any more (Supplementary Fig. S1) and thus differ clearly from the scores in Figure 7. By contrast, the last pair of scores are relatively similar for the observed data and also for the phylogenetically corrected data, indicating that our data indeed contained phylogenetic signal and that this signal was concentrated in the first two K-components. Despite the relatively small number of taxa, the eigenvectors were stable for the range of 5–9 PCs. With a larger number of PCs, the results became increasingly noisy and overfitted (see Supplementary Fig. S2).

**Figure 8:**
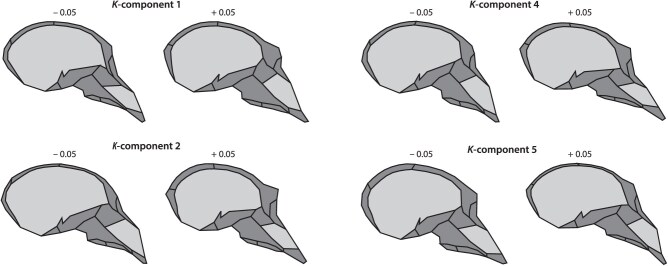
Visualization of the shape patterns corresponding to the 2 components with maximal phylogenetic signal (components 1 and 2) and the 2 components with minimal phylogenetic signal (components 4 and 5).

The shape patterns corresponding to the loadings of the K-components can be visualized just like standard PCs by adding a multiple of the loading vectors (here ±0.5) to a reference shape (here the overall mean shape; Fig. 8). The first 2 components with maximal phylogenetic signal mainly represented variation in the position of the sutures in the anterior cranial base, the naso-frontal complex, and the cranial vault, along with variation in the size of the supraorbital torus. The 2 components with minimal phylogenetic signal represented variation in the orientation of the cranial base and overall facial and neurocranial form. These results mirror the findings of Grunstra et al. (2021), showing that phylogenetic signal is more clearly present in small-scale shape features and structural “details,” such as the sutural patterns, that are likely of little functional relevance. Thus hidden from selection, these features are largely subject to evolutionary drift and better reflect phylogenetic history than more functionally relevant, often large-scale features such as overall cranialform.

## Discussion

Due to the hierarchical structure of the tree of life, it is typically expected that more closely related species resemble each other more than distantly related species; a pattern termed phylogenetic signal. While phylogenetic signal is commonly observed in univariate traits, evaluating whether multivariate phenotypes display similar macroevolutionary trends is challenging because different trait dimensions may display differing degrees of phylogenetic signal. Here, we introduced a new approach for evaluating phylogenetic signal that is capable to exploring how, and to what extent, phylogenetic signal is concentrated in one or a few dimensions. The approach estimates a series of components—the K-components—that describe decreasing levels of phylogenetic signal via linear combinations of the original variables, based on an eigenanalysis of the phylogenetic signal matrix, **K** (a multivariate generalization of Blomberg’s *K*).

The loadings of the K-components can be biologically interpreted (in our examples as anatomical shape deformations; Fig. 8), and scores along the components serve as an ordination of the measured data that maximally or minimally carries phylogenetic signal (first and last K-components, respectively; cf. Figs. 4 and 7). The K-component scores can also be used as variables for further analysis, for example, in functional and ecological analyses, if one wishes to focus on trait dimensions with different phylogenetic signal (e.g., to assess adaptive speciation versus parallel evolution). However, in contrast to PCs, the K-components are not orthogonal (but uncorrelated) and the scores thus do not preserve the Euclidean geometry of data space (distances, angles, total variance) if that has been interpretable for the raw variables (which is not often the case; see Mitteroecker and Huttegger, 2009; Huttegger and Mitteroecker, 2011). For many multivariate statistics, including Mahalanobis distances, Hotelling’s $T^{2}$, MANOVA, linear and quadratic classification, canonical variate analysis, and relative eigenanalysis, the results are the same whether computed from PCs or from K-components computed from these PCs (because all these statistics are affine invariant). The same invariance holds for multiple regressions and derived statistics if the components serve as independent variables. As dependent variables, regression results are affine equivariant, that is, they scale accordingly.

Our approach resolves why some multivariatedatasets yield significant phylogenetic signal, but at much levels lower than those expected under Brownian motion. Many prior studies have attributed this paradoxical result as representing “significant, but weak” phylogenetic signal (discussed in Adams and Collyer, 2019; Collyer and Adams, 2021), implying that levels of phylogenetic signal are low and relatively uniform across trait dimensions. Whereas previous approaches (e.g., $K_{m\,u\,l\,t}$) were incapable of discerning such a pattern, our procedure can show whether the eigenvalues of **K** are indeed relatively uniform, thereby identifying weak, but consistent phylogenetic signal (e.g., Fig. 1c), or whether phylogenetic signal is concentrated in a few trait dimensions (e.g., Fig. 1d).

We showed how null hypotheses of complete phylogenetic independence and of uniform phylogenetic signal can be tested by a permutation approach, which can be useful in determining whether levels of phylogenetic signal within a dataset require biological interpretation. The permutation procedure also gives rise to Z-scores as standardized measures of statistical power for the test statistics. This way, we could show that $K_{A}$ and $K_{G}$ have higher power than $K_{m\,u\,l\,t}$, both for uniform and concentrated phylogenetic signal.

One important implication concerns the calculation of the likelihood describing the fit of multivariate data to the phylogeny under a particular evolutionary model (e.g., Brownian motion). This likelihood may also incorporate the degree of phylogenetic signal by scaling the off-diagonal elements of Ω by λ (see Supplementary Material). Current multivariate extensions of phylogenetic likelihood are based on Revell and Harmon (2008), who used a Kronecker tensor product between the trait covariance matrix and the phylogenetic covariance matrix to incorporate multiple traits into the likelihood equation of Felsenstein (1973). This multivariate formulation uses a single λ for all p trait dimensions, implying that the degree of phylogenetic signal is uniform across the space. Yet we showed that phylogenetic signal can be concentrated in one or a few trait dimensions, which has major implications for how one might best calculate the likelihood describing the fit of multivariate phenotypes to the phylogeny. Several solutions can be envisioned (see Supplementary Material and Collyer et al., 2022), and future work should explore the efficacy and evolutionary interpretation of theseimplementations.

Many multivariate datasets, including our example datasets, require dimension reduction prior to KCA because stable results require full rank data and a clear excess of cases over variables. Prior reduction of the data to a few PCs is effective but involves a somewhat subjective decision about the number of PCs to include. When repeating the example analyses with different numbers of PCs, we found that most of the phylogenetic signal was collectively captured by the first few PCs. The K-components, the resulting ordinations as well as the P-values for the statistics $K_{A}$ and $K_{G}$ were stable for a range of included PCs, whereas the measure of anisotropy ($K_{A}/K_{G}$) and its P-value showed a stronger dependence on dimension reduction (mostly because $K_{G}$ is strongly influenced by small eigenvalues).

Finally, our empirical examples revealed statistically significant and anisotropic phylogenetic signals in crocodyliform and papionin cranial shape. Numerous previous studies tried to identify primate craniodental traits with strong phylogenetic signal, but these approaches compared one cranial region or set oftraits as a whole against others (e.g., Gibbs et al., 2000; Caumul and Polly, 2005; Harvati and Weaver, 2006; Lockwood, 2007; Cardini and Elton, 2008; Smith, 2009; Roseman et al., 2010; von Cramon-Taubadel, 2011; Rathmann et al., 2023). Our approach, by contrast, allows for an exploratory search of linear combinations with high phylogenetic signal of *all* measured traits. As our empirical findings show, phylogenetic signal may be reflected by shape features that are distributed across the entire cranium, such as the relative position of cranial sutures and other “constructional details”; it is not located in any specific region. Future work may show whether or not this is a general pattern in vertebrates. Note that because of its affine invariance, KCA can be applied to various types and combinations of variables at an interval scale, not only geometric morphometric data.

## Acknowledgements

This work was supported by the Austrian Science Fund (FWF) grant P 33736-B (to P.M.) and National Science Foundation grants DEB-2146220 (to M.L.C) and DEB-2140720 (to D.C.A). We thank David Polly and Junja Watanabe for their thoughtful comments on the manuscript.

## Supplementary Material

Data available from the Dryad Digital Repository: https://doi.org/10.5061/dryad.rr4xgxdgk
